# Source Apportionment of PM_**10**_ by Positive Matrix Factorization in Urban Area of Mumbai, India

**DOI:** 10.1100/2012/585791

**Published:** 2012-05-03

**Authors:** Indrani Gupta, Abhaysinh Salunkhe, Rakesh Kumar

**Affiliations:** National Environmental Engineering Research Institute (NEERI), Mumbai Zonal Centre, 89/B Dr. A.B. Road, Worli, Mumbai 400018, India

## Abstract

Particulate Matter (PM_10_) has been one of the main air pollutants exceeding the ambient standards in most of the major cities in India. During last few years, receptor models such as Chemical Mass Balance, Positive Matrix Factorization (PMF), PCA–APCS and UNMIX have been used to provide solutions to the source identification and contributions which are accepted for developing effective and efficient air quality management plans. Each site poses different complexities while resolving PM_10_ contributions. This paper reports the variability of four sites within Mumbai city using PMF. Industrial area of Mahul showed sources such as residual oil combustion and paved road dust (27%), traffic (20%), coal fired boiler (17%), nitrate (15%). Residential area of Khar showed sources such as residual oil combustion and construction (25%), motor vehicles (23%), marine aerosol and nitrate (19%), paved road dust (18%) compared to construction and natural dust (27%), motor vehicles and smelting work (25%), nitrate (16%) and biomass burning and paved road dust (15%) in Dharavi, a low income slum residential area. The major contributors of PM_10_ at Colaba were marine aerosol, wood burning and ammonium sulphate (24%), motor vehicles and smelting work (22%), Natural soil (19%), nitrate and oil burning (18%).

## 1. Introduction

Urbanization has resulted in high levels of ground level deterioration of air quality. The investigation of air pollution in mega cities by Mage et al. [1] showed that the major problem affecting these cities is their high levels of particulate matter (PM). PM is composed of a broad class of chemically and physically diverse substances. It is well established that high levels of PM are significantly associated with adverse health effects, ecosystem damage, and degraded visibility [2]. Health effects associated with PM are linked to respiratory, cardiovascular health problems, and premature mortality [3, 4].

Receptor modeling is the application of multivariate statistical methods addressed to the identification and quantitative apportionment of air pollutants to their sources. During last few years, receptor models have been used effectively for developing air quality management plans in various cities. Different models including principal component analysis/absolute principal component scores (PCA-APCS) [5, 6], edge analysis (UNMIX, [7]), chemical mass balance (CMB) [8], and positive matrix factorization (PMF) [9] have been applied by several researchers to identify and establish the sources contributing to ambient air. UNMIX uses geometrical objects called the edges to identify factors. UNMIX does not allow individual weighing of data points as does PMF. Although major factors resolved by PMF and UNMIX are generally the same, UNMIX does not always resolve as many factors as PMF [10, 11]. With CMB, the user must provide source profiles which the model uses to apportion mass. PMF and CMB have been compared in several studies. Rizzo and Scheff [12] compared the magnitude of source contributions resolved by each model and examined correlations between PMF and CMB-resolved contributions. They found that the major factors correlated well and were similar in magnitude. Additionally, PMF-resolved source profiles were generally similar to measured source profiles. Recently, Callén et al. [13] carried out source apportionment of PM_10_ in Zaragoza, Spain by three multivariate receptor models based on factor analysis: PCA-APCS, UNMIX, and PMF. Special attention was paid to the models comparison in order to determine which models were more adequate for the apportionment. They concluded that greater requirements of measure of uncertainty in PMF permitted to obtain better results than with the other two models: PCA-APCS and UNMIX.

Therefore, in this paper, source apportionment of PM_10_ has been carried out using PMF technique. The study makes an attempt to bring out large-scale variability within a city by identifying different sources. The study was performed in the city of Mumbai, India with the monitored samples during April 2007 to March 2008. Mumbai is a coastal city, with three sides surrounded by sea, harbor, and creek, respectively. Its climate is highly influenced by sea and land breeze phenomena along with about four months of extended rainy season between June and September. The city witnesses contribution of air pollution from traffic, industries, re-suspended dusts due to large-scale construction/demolition, and refuse burning. Air quality monitoring was carried out in all the three seasons, namely, summer, postmonsoon, and winter. Ambient concentration data used in the source-receptor modeling include PM_10_ mass, anions and cations, total organic carbon (OC), elemental carbon (EC), and elements.

## 2. Sampling and Chemical Analysis

**Figure 1:**
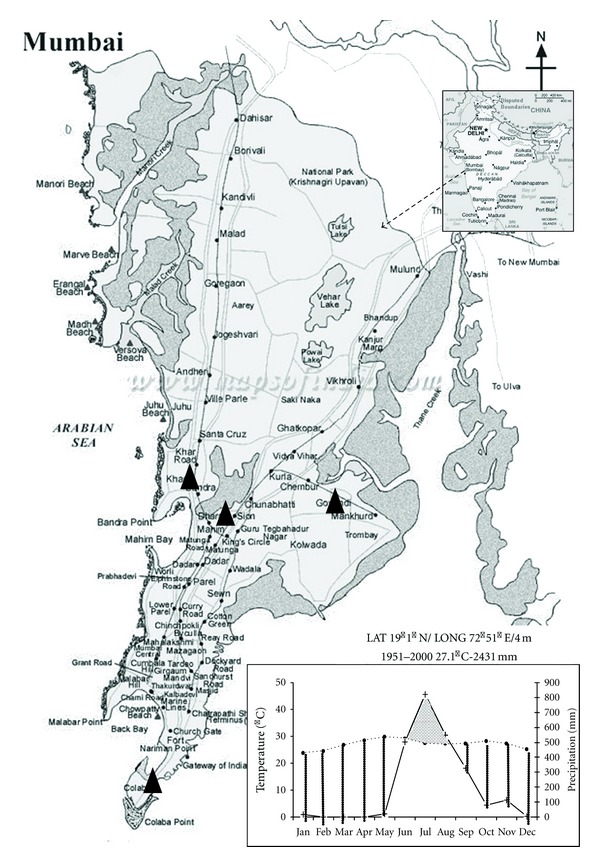
Air-monitoring stations at Mumbai along with the Walter-Liet diagram.

Figure 1 provides the locations of sites within the Mumbai city, along with the Walter-Liet diagram describing the climate of the city. Air quality monitoring was carried out at four sites which included industrial site located at Mahul, residential area of upper income group at Khar, mixed residential site comprising low-income residential, commercial and small-scale units along with industrial at Dharavi, and a background site at Colaba. The first site at Mahul was situated near the petroleum industry complex Bharat Petroleum Corporation Limited and Hindusthan Petroleum Corporation Limited, Indian Oil Blending Ltd. and a chemical fertilizer plant RCF. Tata Thermal Power Plant is located in close proximity of this area. Containers and heavy duty vehicles ply within this area. The residential site at Khar was second site located on S.V. Road, a busy road connecting Southern Mumbai with Western suburbs. This site contributes to the vehicular traffic in this area. Building construction and demolition activities are common around the Khar site; however, it does not have any industrial activities. The third site at Dharavi was within Asia's largest slum conglomerate having a population of more than 1 million. About 15 ,000 single-room factories of small-scale operations mainly glassworks, leathers, plastic pellets, jewellery, small-scale food processing industries, welding operations, and so forth are located within this area. Waste created from these activities is burnt in open area. The fourth site at Colaba represents the “Background Site” for the study region. It has Arabian Sea on one side and residential area on the other side. The southern side is occupied by a military cantonment, including the Navy. No industrial activities are located in this area except a dock area that may have direct contribution of air pollutants.

Samples were collected for summer, postmonsoon, and winter seasons to represent seasonality at each site. In each season, sampling was carried out continuously for one month. Ambient concentration data used in the source-receptor modeling include PM_10_ mass, anions, cations, total organic carbon (OC), elemental carbon (EC), and elements. The analytical methods include gravimetric analysis using a microbalance, ionic analysis using Ion Chromatograph, trace metal analysis using ICP-AES, and elemental carbon and organic carbon analysis using DRI thermal/optical carbon analyzer.

Ambient air sampling of PM_10_ at four sites was carried out using Partisol Model 2300 Speciation Sampler of Rupprecht and Patashnick Co., USA. The system is designed to collect samples using four channels simultaneously each with the set flow rate of 16.7 ± 0.1 lpm. PM_10_ Samples were collected on two different filter media. PM_10_ mass collection was carried out with two PTFE ring supported Teflon filters (size of 47 mm and 1 *μ* pore size, Schindeler Whatman, USA). One pure tissue quartz filter (size of 47 mm and 1 *μ* porosity, Pall Life Sciences Co. USA) was also used during each day of sampling per site.

Weighing was carried out on an electromicrobalance with 1*μ*g sensitivity (Sartorius Model ME 5-F). Unexposed and exposed Teflon membrane filters were kept in a temperature and humidity-controlled clean room (temperature 20°C ± 3°C and 40% ± 5% RH). Each filter was weighed in duplicate for getting average weight of exposed and unexposed filters.

Particle mass collected on tissue quartz paper was analyzed for OC and EC using Desert Research Institute's Thermal/Reflectance Optical Carbon Analyzer (model DRI2001, Protocol Improve A). The analysis is based on liberating carbon compounds at different temperatures. During the analysis, correlation coefficient greater than 0.995 was maintained.

For water soluble inorganic ion analysis, PM_10_ samples collected on Teflon filters were subjected to ultrasonic extraction with the help of ultra pure water having the conductivity about 18 Ω. Water extract was then subjected to filtration using Teflon syringe filter with porosity 0.45 *μ*m and the samples were ready for analysis. Finally the samples were analyzed by Ion Chromatograph (Dionex Corporation US Model ICS3000).

In present study, trace elements were estimated from PM_10_ samples collected on Teflon filters using inductive couple plasma-atomic emission spectroscopy (Model-Horiba Jobin-Yvon, Ultima 2000). The extraction was performed by adding aqua regia. National Institute of Standards and Technology (NIST) traceable certified standards were used for preparing the calibration standards.

## 3. Model Description of EPA PMF 3.0

Positive matrix factorization PMF is a powerful multivariate technique that constraints the solution to be nonnegative and takes into account the uncertainty of the observed data [9]. This method relies on the time invariance of the source profiles and, thus, requires the emission particle size distributions to be stable in the atmosphere between the sources and the receptor site. It is reasonable to expect that particle size distributions will become relatively stable when sampling is carried out at some appropriate distance from the emission sources after initial size distribution changes in the vicinity of the sources due to coagulation and dry deposition [14].

A speciated data set can be viewed as a data matrix *X* of *n* by *m* dimensions, in which *n* is number of samples and *m* is chemical species to be measured. The goal of multivariate receptor modeling is to identify the number of factors *p*, the species profile *F*(*p* × *m*) of each source, and the amount of mass *G*(*n* × *p*) contributed by each factor to each individual sample.

The model solves the general equation

(1) X=G·F+E,

where *E* is the residual matrix (observed estimated).

Equation (1) can also be expressed in the element form as

(2) $$x_{ij}=\overset{p}{\underset{k=1}{\sum }}g_{ik}f_{kj}+e_{ij},$$

where, *x*_*ij*_ is the *j*th elemental concentration measured in the *i*th sample, matrix *g*_*ik*_ is the fraction of total PM_10_ concentration from source *k* in sample *i*, *f*_*kj*_ is the gravimetric mass of each element *j* per unit PM mass emitted from each source *k*, and *e*_*ij*_ is the residual for each sample/species. The objective is to find *G* and *F* by minimizing the residual error *E*. Further the elements of *F* and *G* are constrained to be nonnegative. For this a weighted least square approach is used. It involves minimization of an objective function *Q*, given as

(3) $$\begin{array}{cc} \text{minimize} & \;Q=\overset{n}{\underset{i=1}{\sum }}\;\overset{m}{\underset{j=1}{\sum }}\frac{e_{ij}^{2}}{s_{ij}^{2}} \end{array}$$

(4) $$\begin{array}{cc} \text{subject}\;\text{to} & \;g_{ik}\geq 0,\;f_{kj}\geq 0, \end{array}$$

where *s*_*ij*_ is an uncertainty estimate in the *j*th species measured in the *i*th sample. The solution of (3) is obtained using an iterative minimization algorithm. Multilinear engine (ME-2) is the underlying program used to solve the PMF 3.0 problem in the program EPA PMF. ME-2 performs the iterations via the conjugate gradient algorithm until convergence to a minimum *Q* value. The minimum *Q* may be global or local. A user has to determine the global minimum by using different starting points for the iterative process and comparing the minimum *Q* value reached. The differences in ME-2 and PMF2 have been examined by several researchers by the application of each model to the same data set. Overall, the studies showed similar results for the major components, but a greater uncertainty in the PMF2 results [15] and better source separation using ME-2 [16].

### 3.1. Uncertainty Calculation

Several researchers [17–19] have estimated uncertainties in the measurement dataset using analytical uncertainty *s*_*ij*_ and adding 1/3rd of the method detection limit (DL_*ij*_) to it as

(5) $$\begin{array}{c} \text{Uncertainty}\;\sigma_{ij}=s_{ij}+\frac{{\text{DL}}_{ij}}{3}. \end{array}$$

Analytical uncertainty *s*_*ij*_ is calculated as a function of concentration

(6) $$s_{ij}=\sigma_{B}+M\sigma_{\text{REL}},$$

where *σ*_*B*_ is standard deviation of laboratory blanks in micrograms, and *M* is analytical mass (micrograms/filter). Relative uncertainty multiplier *σ*_REL_ is used to account for flow/volume variability and handling artifacts such as variability in temperature and humidity while transport from field to laboratory. RTI [20] suggests the use of *σ*_REL_ of 5% of concentration for all analytes and all instruments. Equations (5) and (6) were used here for anions, cations and elements. Method detection limits (MDLs) are based on analytical replicates (usually blanks) and do not include a component of field variability. The MDL values are taken as 3 *σ* values although the method of determining MDL varies with the analysis method. MDLs do not take into account any bias that may be present. 

When the measured sum of chemical species is not close to measured PM, the PM time series would provide additional information to PMF model. Hence in such cases, PM is included as an explicit species with a large uncertainty of four times the concentration [10, 21]. In this study, mass balance closure was tested by comparing the measurements of gravimetric mass and sum of chemical species. Gravimetric PM mass was higher than the sum of measured species at all sites. The difference between Gravimetric PM_10_ mass and sum of measured species varied between 18 and 43%. Hence, PM_10_ was included in the PMF concentration matrix. Additional PMF runs when excluded PM as a species had drawbacks such as highly mixed factors and physically unrealistic factors. In comparison, solutions including PM_10_ as an explicit species gave superior factor resolution and more realistic factor composition.

The variables or species to be included in the PMF analysis were selected using the signal to noise ratio.

The *S*/*N* ratio is defined as

(7) $${\left(\frac{S}{N}\right)}_{j}=\frac{\sum_{i=1}^{n}{\left(x_{ij}-s_{ij}\right)}^{2}}{\sum_{i=1}^{n}{\left(s_{ij}\right)}^{2}}.$$

A variable is called “weak” if the *S*/*N* ratio is between 0.2 and 2. Variables with *S*/*N* ratio less than 0.2 are denoted as “bad” variables and are excluded from the analysis.

### 3.2. Goodness-of-Fit Parameters

The *Q* values are goodness-of-fit parameters calculated using (3) and are an assessment of how well the model fit the input data. *Q*_robust_ is calculated excluding outliers, defined as samples for which the scaled residual is greater than 4, and the *Q*_true_ is calculated including all points. Solutions where *Q*_true_ is greater than 1.5 times of *Q*_robust_ indicate that peak events may be disproportionately influencing the model. The model was run based on a user specified number of factors and number of iterations. Subsequent to the model run, the model calculated *Q*_robust_ values for each random run are compared to *Q*_theoretical_ values to check the model performance.

Selection of the number of factors or sources is subjective. The user must select a maximum number of factors that can adequately describe the total PM_10_ mass while excluding factors that do not make physical sense, such as duplicate factors or factors with unrealistic compositions or contributions. Knowledge of the possible sources in the area is crucial as it can provide an answer where factors do not show clear separation. Evaluating multiple solutions within the range of *F*_peak_ values that yield an acceptable *Q* value and assessing the edge plots are more objective ways to evaluate the model results

### 3.3. *F*_peak_ Runs

A pair of factor matrices (*G* and *F*) that can be transformed to another pair of matrices (G* and F*) with the same *Q* value is said to be “rotated”. The transformation takes place as follows:

(8) $$G^{*}=GT,\;\;F^{*}=T^{-1}F.$$

The *T* matrix is a *p* × *p*, nonsingular matrix. In PMF, this is not strictly a rotation but rather a linear transformation of the *G* and *F* matrices. Due to the nonnegativity constraints in PMF, a rotation (i.e., a specific *T* matrix) is only possible if none of the elements of the new matrices are less than zero. If no rotation is possible, the solution is unique.

In EPA PMF 3.0, the base model run with the lowest *Q*_robust_ is automatically selected by the program as the base run for *F*_peak_ runs. The user can perform up to five *F*_peak_ runs by checking the appropriate number of boxes and entering the desired strength of each *F*_peak_ run. Generally values between −5 and 5 should be explored first although there are no limits on the values that can be entered as *F*_peak_ strengths. Positive *F*_peak_ values sharpen the *F* matrix and smear the *G* matrix, and negative *F*_peak_ values smear the *F* matrix and sharpen the *G* matrix. PMF2 [22] was originally used to solve PMF model. Multilinear engine version 2 (ME-2) is the underlying program used to solve the program EPA PMF 3.0 [23]. The *F*_peak_ strengths in ME-2 are not the same as those in PMF2; values of around 5 times the PMF2 values are needed to produce comparable results in ME-2. Additionally, an *F*_peak_ value of 0 is not allowed.

## 4. Source Identification Using Positive Matrix Factorization

**Figure 2:**
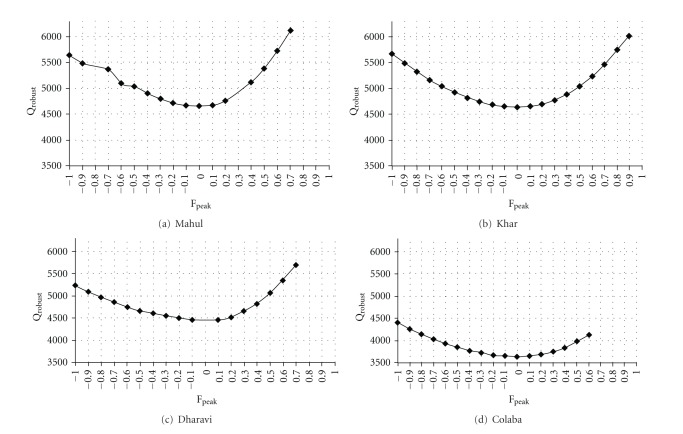
Position of original solution using different values of *F*_peak_ for all sites.

**Figure 3:**
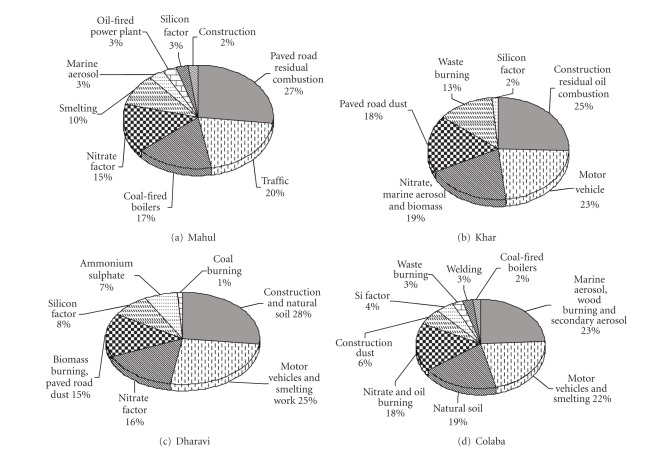
Source contribution from four monitoring sites.

**Figure 4:**
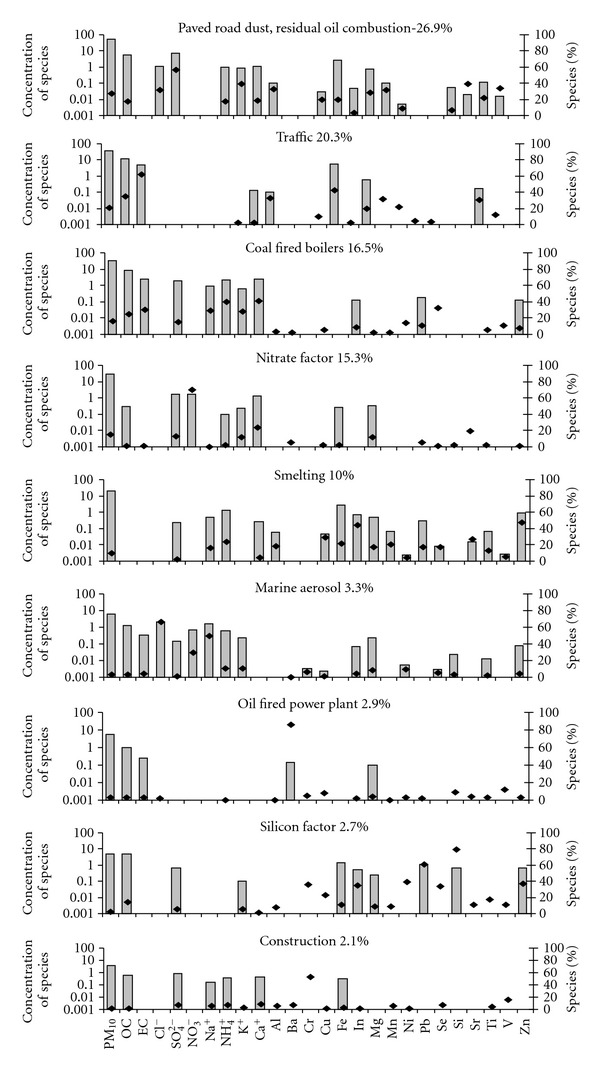
PMF factor Profile for Mahul.

**Figure 5:**
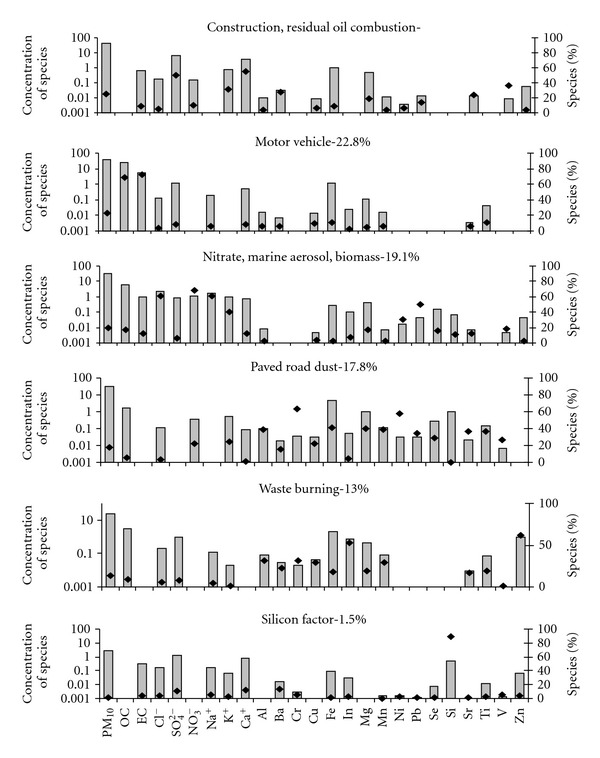
PMF factor profile for Khar.

**Figure 6:**
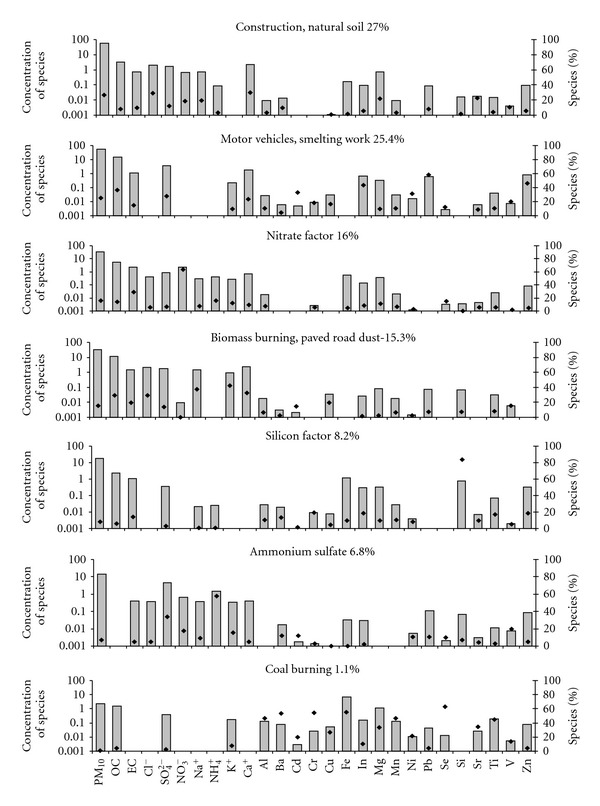
PMF factor profile for Dharavi.

**Figure 7:**
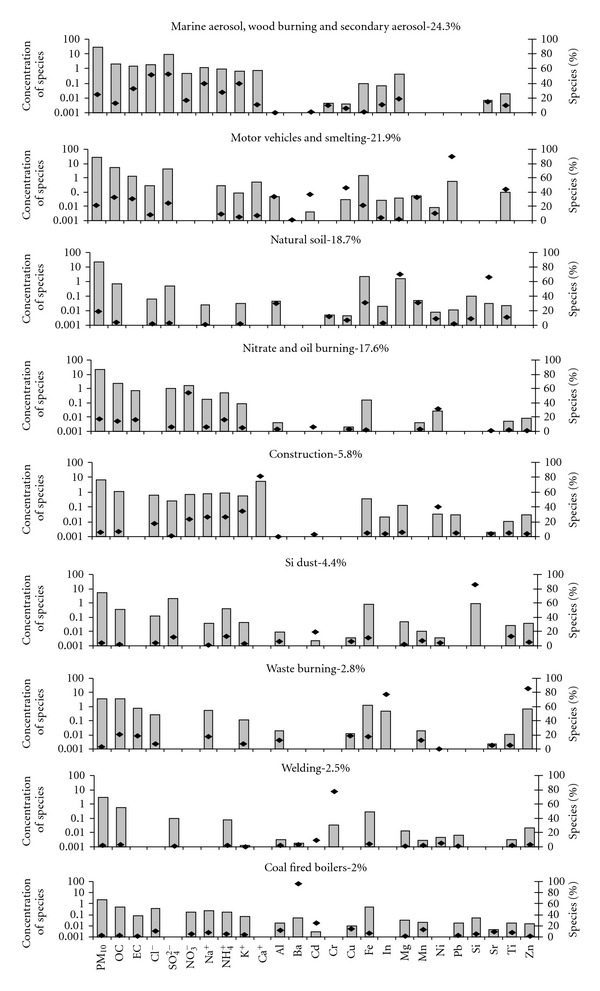
PMF factor profile for Colaba.

Sodium (Na), potassium (K), chloride (Cl), calcium (Ca), and magnesium (Mg) were included in their ionic form, but their elemental form (measured by ICPAES) was excluded to avoid double counting of mass [24]. Selenium (Se) and Vanadium (V) were not detected at Colaba, and Cadmium (Cd) was not detected at Khar.  Table 1 shows the concentrations with standard deviations of PM_10_ and the identified species at four sites. The number of species used for PMF modeling ranged from 24 to 26. The number of valid samples at four sites used for modeling ranged between 83 and 86. To identify the likely number of factors, 20 random runs were used and the run with the minimum estimated Q value was retained [23, 25] for 5–10 factors. Reduction in *Q* with increase in number of factors and agreement of estimated *Q* with its theoretical value *Q*_theoretical_ were used to identify probable solutions. Estimated *Q* decreased with increasing number of factors. Solutions of 6, 7, 8, and 9 factors were carefully examined. Source categories were recognized from the PMF factor profiles based on the abundance of one or several tracer species. Rotation of the original solution using different values of *F*_peak_ is shown in Figure 2. *F*_peak_ solutions gave a higher loading of tracer species in many factors and resulted in the increase in the number of zeros in the G or *F*-matrix, while the *Q*_robust_ value increased. The physical interpretation of factors in terms of likely source categories was the main criterion used for the choice of base result or result obtained by *F*_peak_. The *Q*_theoretical_ values for Mahul, Khar, Dharavi, and Colaba were 2125, 2064, 2158 and 2064 respectively. The minimized *Q*_robust_ values for Mahul, Khar, Dharavi, and Colaba were 4755, 4432, 4636, and 3685, respectively. *Q*_true_ was found to be within 1.5 times the *Q*_robust_. Several studies report similar variations in the *Q*_robust_ values as compared to the *Q*_theoretical_ values for PMF model runs. Kim et al. [26] report *Q*_robust_ value of 4424 against the *Q*_theoretical_ value of 2369. Average contribution of each factor to PM_10_ mass at the monitoring sites is given in Figure 3. The choice of tracers, for a source category identified in this study, has been discussed in the description of the factors for each site. The PMF factor profiles for Mahul, Dharavi, Khar, and Colaba are given in Figures 4, 5, 6, and 7.

### 4.1. Description of PMF Sources

#### 4.1.1. Mahul

Optimal number of factors chosen was 9. A comparison of the daily mean reconstructed PM_10_ concentrations from all sources with the measured PM_10_ concentrations shows that the identified factors effectively reproduce the measured mass and account significantly for the variation in the PM_10_ concentrations (*R* ^2^ = 0.87). Species with *R* ^2^ > 0.9 were Cl^−^, NH_4_^+^, Ca^2+^, Al, Ba, Cr, Cu, Fe, In, Mg, Mn, Pb, Si, Sr, Ti, and Zn. Species with *R* ^2^ > 0.8 were OC, SO_4_^2−^, NO_3_^−^, Na^+^, Ni, and Se.

The first factor was dominated by SO_4_^2−^, Al, Mn, K^+^, Sr, and V. SO_4_^2−^ and V are tracers for residual oil combustion [27, 28]. Al, Mn, K, and Sr are tracers for paved road dust. The contribution of this factor was 26.9% of PM_10_ mass on an average at Mahul.

The traffic factor contributes 20% of PM_10_ mass on an average at Mahul. The components of this factor were OC, EC, Al, Fe, and Mg. 62% of total EC, and 35% of total OC is present in this factor. These species are tracers for traffic [27, 29].

The coal-fired boiler factor contributed 16.5% of the PM_10_ mass on an average. OC, EC, NH_4_^+^, Ca^+^, and Se are the species which dominate this factor. These species are tracers for coal-fired boiler [27]. The Tata thermal power plant is located within a distance of about 3 km.

The nitrate factor contributes 15.3% of the PM_10_ mass on an average. 70% of nitrate is present in this factor. The major source of nitrate is the conversion of nitrogen oxides (NOx) emitted from high-temperature combustion sources making this category predominantly secondary material.

Cu, In, and Zn are dominant in this factor which explains 10% of the PM_10_ mass. These are tracers of smelting [30].

PMF model identified 4 more factors. The marine aerosol factor contributes 3.3% of the PM_10_ mass. The Ba factor contributes 2.9% of the PM_10_ mass. 63% of total Ba is present in this factor. Ba is a tracer for oil-fired power plant [27]. The silicon factor contributes 2.7% of the PM_10_ mass. Bhanuprasad et al. [21] also reported a Si factor (20–30%) from PMF analysis that influenced surface concentrations of aerosols in the Indian Ocean Experiment (INDOEX), measured onboard with probable source regions from potential source contribution function (PSCF). The Cr factor contributes 2.1% of PM_10_ mass and may be from construction activities [27].

#### 4.1.2. Khar

The coefficient of determination between daily mean reconstructed PM_10_ concentrations from all sources and the measured PM_10_ concentrations was 0.87. It shows that the identified factors effectively reproduce the measured mass and account significantly for the variation in the PM_10_ concentrations. Species with *R* ^2^ > 0.8 were found to be OC, EC, SO_4_^2−^, K^+^, Ca^2+^, Al, Cu, Cr, Fe, In, Mg, Mn, Pb, Si, Sr, Ti, V, and Zn. Cd was not detected at Khar. A total of 6 factors were chosen as the optimal number for the PMF model.

The first factor was dominated by Ca^2+^, SO_4_^2−^, and V. V and SO_4_^2−^ are tracer species for residual oil combustion [27]. Calcium is tracer species for construction [27]. The contribution of this factor was 25.4% of the PM_10_ mass on an average at Khar.

The motor vehicles factor contributes 22.8% of total PM_10_. 68.4% of total OC and 72.7% of total EC are present in this factor. EC and OC are tracers for motor vehicles [27, 30]. The sampling site was adjacent to a major road, S.V. Road.

The third factor was dominated by the presence of Na^+^, Cl^−^, and NO_3_^−^. This factor contributes 19.1% to the PM_10_ mass on an average. 60.6% of soluble Na and 60.7% of soluble Cl is present in this factor. 67.7% of the total NO_3_^−^ is also present in this factor. The model could not distinguish between marine aerosol and Nitrate at Khar.

The next factor was dominated by Cr, Mn, Al, Ti, Sr, P and Ni. These are tracers for paved road dust [27]. The contribution of paved road dust was 17.9% to the PM_10_ mass on an average at Khar.

Another factor was dominated by Zn and In. Zn is a tracer for waste burning [30]. This factor also comprises of Cr, Al and Mn which are tracers of natural soil [27]. Waste burning and Natural soil contribute 13% of total PM_10_.

#### 4.1.3. Dharavi

Optimal number of factors chosen was 7. The coefficient of determination between daily mean reconstructed PM_10_ concentrations from all sources and the measured PM_10_ concentrations was 0.6. It shows that the identified factors reproduced the measured mass reasonably well. Species with *R* ^2^ > 0.9 were Cl^−^, Na^+^, Al, Ba, Cr, Cu, Fe, In, Mg, Mn, Pb, Si, Sr, Ti, and Zn. Species with *R* ^2^ lying between 0.8 and 0.9 were SO_4_^2−^, NO_3_^−^, NH_4_^+^, K^+^, Ca^2+^, Cd and Ni.

About 30% of Ca^2+^ and 23% of Sr dominated the first factor. Ca^2+^ and Sr are tracers for construction work [27]. 29% of Cl^−^ and 22% of Mg^2+^ were also present in this factor, which are tracers for Natural soil [27]. The Model could not distinguish between these sources. The contribution of construction dust and natural dust was 27% of PM_10_ mass on an average at Dharavi and was found to be the largest contributor to PM_10_ at this site.

The second factor was dominated by Zn, OC, EC, and SO_4_^2−^. These species are tracers for motor vehicles [27]. In India Pb free gasoline is being used and PB does come from motor vehicles. About 60% of total Pb, 43.1% of total In and 33.3% of Cd is also present in this factor which are tracer species for Smelting works [30]. The contribution of this factor was 25.4% of PM_10_ mass and was the second largest contributor to PM_10_ at this site.

The third factor was dominated by the presence of nitrate and its contribution was 16% to the PM_10_ mass on an average at Dharavi. 64% of total nitrate was observed in this factor.

The fourth factor was dominated by 32.4% of Ca^2+^, 30% of Cl^−^ and 29% of OC. These are tracers for paved road dust. 42% of total K^+^ was also present in this factor which is a tracer for biomass burning [21]. The total contribution of this factor was 15.3% of the total PM_10_ mass.

Apart from above four factors, PMF model identified 3 more factors. A silicon factor contributing 8.2% of total PM_10_ mass was also resolved by the model. Sulphate factor was dominated by the presence of sulphate and ammonium and contributed 6.8% of total PM_10_ mass. The presence of several small-scale industries in the vicinity of Dharavi validates the high sulphate contribution. The seventh factor contributed only 1.1% of total PM_10_ mass. This factor was composed mainly of Se, Al, Mn, Ti, Fe, and Ba which are tracers of coal fired boiler. The presence of several unauthorized small-scale industries within Dharavi validates this factor.

#### 4.1.4. Colaba

Optimal number of factors chosen was 9. A comparison of the daily mean reconstructed PM_10_ concentrations from all sources with the measured PM_10_ concentrations shows that the identified factors effectively reproduce the measured mass and account significantly for the variation in the PM_10_ concentrations (*R* ^2^ = 0.82). It shows that the identified factors reproduced the measured mass well accounting reasonably for the variation in the PM_10_ concentrations. Species with *R* ^2^ > 0.9 were SO_4_^2−^, Ca^2+^, Al, Ba, Cr, Cu, Fe, In, Mg^2+^, Mn, Pb, Si, Sr, Ti, and Zn. Species with *R* ^2^ lying between 0.7 and 0.9 were NO_3_^−^, Ni and Na^+^. Selenium (Se) and Vanadium (V) were not detected at Colaba.

The first factor was dominated by Na^+^, Cl^−^, SO_4_^2−^, NH_4_^+^, K^+^, and EC. 39% of Na^+^ and 51% of Cl^−^ representing the marine aerosol was present in this factor. Colaba is a site surrounded on three sides by sea. In urban areas, the possible sources of secondary sulphate include fuel combustion in vehicles and coal-fired power plants. Marine aerosol is also a source for sulphate [27]. 39.7% of K^+^ which is a tracer species for wood burning was also present [31]. In Colaba there are several bakeries that use wood as the fuel, and may be a possible source for K^+^. The PMF model could not resolve the three sources, namely, marine aerosol, secondary aerosol and wood burning. The average PM_10_ mass contribution of this factor was 24.3% at Colaba.

The important components of second factor were EC (30%), OC (33%), Pb (90%), Cu 46(%), Mn (33%), and Al (33%). These species are tracers for motor vehicles [27]. Apart from these, Ti (44%) and Cd (36%) were also observed. Cu, Pb, Cd, and Ti are tracers of smelting [27]. The contribution of this factor was 21.9% of PM_10_ mass on an average at Colaba. The PMF analysis does not distinguish very well between these two sources.

The third factor was dominated by the presence of Mg^2+^ (70%), Sr (66%), Mn (31%), Fe (31%), and Al (30%). This factor contributed 18.7% to the PM_10_ mass on an average at Colaba and can be attributed to natural soil as Mg, Sr, Mn, Fe, and Al are considered its tracers [27].

About 17.6% of PM_10_ mass at Colaba comprised of 53.7% of NO_3_^−^ and 31.3% of Ni in the fourth factor. Ni is a tracer of burning of residual fuel oil [32].

The fifth factor had 81.3% of Ca^2+^ which contributed 5.8% of PM_10_ mass at Colaba. Calcium is a tracer for construction dust [27]. The next factor accounted for 4.4% of PM_10_ mass with 86% of Si presence. The next factor accounted for 2.8% of PM_10_ mass and comprised of Zn, In, Cu, OC, and EC. Zn, In, Cu, OC, and EC are tracers for waste burning [30]. The model identified two more factors which accounted for 2.5 and 2% of PM_10_ mass, respectively. Cr (77.5%) dominated the first factor and was identified due to welding activities in nearby docks present in this area. The second factor was dominated by Ba (96%) and can be attributed to oil fired power plant [27]. Some major refineries are located at a distance of about 13 km and possible source of contribution.

## 5. Conclusions

EPA PMF3.0 was used to analyze the elemental data obtained from four sites in Mumbai. The number of sources varied between 6 and 9. The major contributors of PM_10_ at the industrial site Mahul were residual oil combustion and paved road dust (26.9%), traffic (20.3%), coal-fired boiler (16.5%), nitrate (15.3%), and smelting (10%). Khar, a residential area of upper income group received contributions from residual oil combustion and construction (25.4%), motor vehicles (22.8%), marine aerosol and nitrate (19.1%), paved road dust (17.9), and waste burning and natural soil (13%). At Dharavi, Asia's largest slum conglomerate, major air pollution sources were identified as construction and natural dust (27%), motor vehicles and smelting work (25.4%), nitrate (16%), and biomass burning and paved road dust (15.3%). The major sources at the background site, Colaba, were marine aerosol, wood burning and ammonium sulphate (24.3%), motor vehicles and smelting work (21.9%), natural soil (18.7%), and nitrate and oil burning (17.6%). Findings indicate that most of the sites were dominated by local sources based on activities in the vicinity of the sampling locations. Overall action plan preparation will need to concentrate on local sources as priority, as reduction of these source strengths will give maximum benefit in terms of lower exposure from air pollution.

## Figures and Tables

**Table 1:** Mean and standard deviations (elemental concentration in *μ*g m^−3^) of species of four sites of Mumbai.

	Mahul		Khar		Dharavi		Colaba	
	Average	Std. dev	Average	Std. dev	Average	stdev	Average	stdev
	85 samples		86 samples		83 samples		86 samples	
PM_10_	199.228	83.324	184.930	98.427	230.974	74.980	139.785	75.721
OC	37.111	20.843	36.725	26.855	46.437	22.932	19.875	9.659
EC	9.091	5.971	8.444	6.706	10.065	5.413	6.035	3.845
Cl^−^	3.312	1.922	4.568	3.194	7.303	4.111	4.053	2.110
SO_4_^2−^	12.679	6.093	13.226	8.309	13.385	5.496	16.846	7.786
NO_3_^−^	2.380	1.838	1.987	1.101	3.917	2.623	2.997	2.110
Na^+^	3.165	1.643	3.248	1.918	4.020	2.298	3.014	1.196
NH_4_^+^	5.692	2.951			2.628	1.205	3.961	1.468
K^+^	2.196	0.706	2.420	0.618	2.381	1.122	1.799	1.005
Ca^2+^	5.676	2.155	6.426	3.645	7.846	2.679	6.582	5.957
Al	0.310	0.212	0.270	0.194	0.277	0.191	0.155	0.147
Ba	0.165	0.333	0.143	0.107	0.146	0.112	0.057	0.063
Cd	0.019	0.019			0.015	0.010	0.012	0.010
Cr	0.060	0.041	0.069	0.062	0.051	0.045	0.041	0.069
Cu	0.159	0.116	0.157	0.118	0.190	0.135	0.070	0.052
Fe	13.020	9.445	11.407	7.765	11.764	8.472	7.247	6.556
In	1.533	1.233	1.396	1.040	1.658	1.202	0.635	0.580
Mg	2.883	1.333	2.422	1.394	3.330	1.730	2.313	3.723
Mn	0.318	0.220	0.277	0.200	0.284	0.196	0.158	0.152
Ni	0.064	0.041	0.061	0.026	0.052	0.039	0.087	0.049
Pb	1.804	1.390	0.107	0.043	1.100	0.772	0.677	0.588
Se	0.053	0.035	0.979	0.591	0.023	0.024		
Si	0.831	0.808	0.609	0.676	0.931	1.509	1.030	0.975
Sr	0.055	0.028	0.058	0.035	0.076	0.044	0.048	0.068
Ti	0.552	0.352	0.397	0.291	0.423	0.321	0.215	0.135
V	0.060	0.045	0.027	0.011	0.046	0.055		
Zn	1.857	1.512	1.595	1.243	1.759	1.362	0.779	0.693
